# Do open data impact citizens’ behavior? Assessing face mask panic buying behaviors during the Covid-19 pandemic

**DOI:** 10.1038/s41598-022-22471-y

**Published:** 2022-10-20

**Authors:** Yuya Shibuya, Chun-Ming Lai, Andrea Hamm, Soichiro Takagi, Yoshihide Sekimoto

**Affiliations:** 1 Center for Spatial Information Science, The University of Tokyo, Tokyo, Japan; 2 Department of Computer Science, Tunghai University, Taichung City, Taiwan; 3 Department for Electrical Engineering and Computer Science, Technical University Berlin, Berlin, Germany; 4 Interfaculty Initiative in Information Studies, The University of Tokyo, Tokyo, Japan

**Keywords:** Civil engineering, Health policy

## Abstract

Data are essential for digital solutions and supporting citizens’ everyday behavior. Open data initiatives have expanded worldwide in the last decades, yet investigating the actual usage of open data and evaluating their impacts are insufficient. Thus, in this paper, we examine an exemplary use case of open data during the early stage of the Covid-19 pandemic and assess its impacts on citizens. Based on quasi-experimental methods, the study found that publishing local stores’ real-time face mask stock levels as open data may have influenced people’s purchase behaviors. Results indicate a reduced panic buying behavior as a consequence of the openly accessible information in the form of an online mask map. Furthermore, the results also suggested that such open-data-based countermeasures did not equally impact every citizen and rather varied among socioeconomic conditions, in particular the education level.

## Introduction

Open data expanding in cities and communities presents new opportunities for better understanding of and communicating in urban spaces^1–4^. In addition, open data is essential for constructing smart cities to enable diverse actors to engage in its processes, making governance more accountable and transparent while accelerating innovative solutions^4–14^. Open data has been defined as the data that can be “freely used, modified, and shared by anyone for any purpose”^15^. In related work, however, it has been highlighted that realizing the promise of data’s benefits into tangible, measurable, and consistent outcomes remains largely elusive^4,16^. Opening up data does not automatically translate into its application to provide a solution^16^. Instead, it may end up remaining as information overload^8–10,17,18^. Thus, prioritizing users’ perspectives and incentivizing people to make actual use of open data by collaborating with various actors are important in the context of smart cities^3,4,8,19–34^. In the current state of research, open data is believed to not reaching its expected level of reuse. Scholars have emphasized that open data usage should be designed for ordinary people and not just for people having a higher technological literacy^35–37^. Creating visually attractive and easy-to-use interfaces while considering the city’s local context is critical to making digital service accessible and valuable for everyone, especially people without advanced technical skills^2,4,8–10,23,29,38–43^. Another reason for the lack of value of open data portals is that information needs of citizens are not always well understood and need further investigation to allow applications accurately addressing these needs^44^. Apart from basic functionalities, many platforms would not deliver more value to users^45^. Hence, it remains an important research objective to better understand how open data portals can be designed to be more valuable for citizens, and not only for experts.

We diagnose that there is a need for evaluations on open data use cases^5,6,41,46^. To fully benefit from open data, cities and communities must look beyond merely tracking the number of published and downloaded data or measuring the level of openness to determine the impacts of open data. Though such indexes provide valuable descriptions of data interoperability, they cannot report how the data is being used and by whom, and how it changes people’s behaviors from an end-user perspective^5,6,41,46,47^. To better understand social benefits of open data, open data initiatives need to be scientifically assessed as case studies. In this regards, the focus should lie on understanding better by whom it is used and to what extent open data impacted lay citizens^48^.

**Figure 1:**
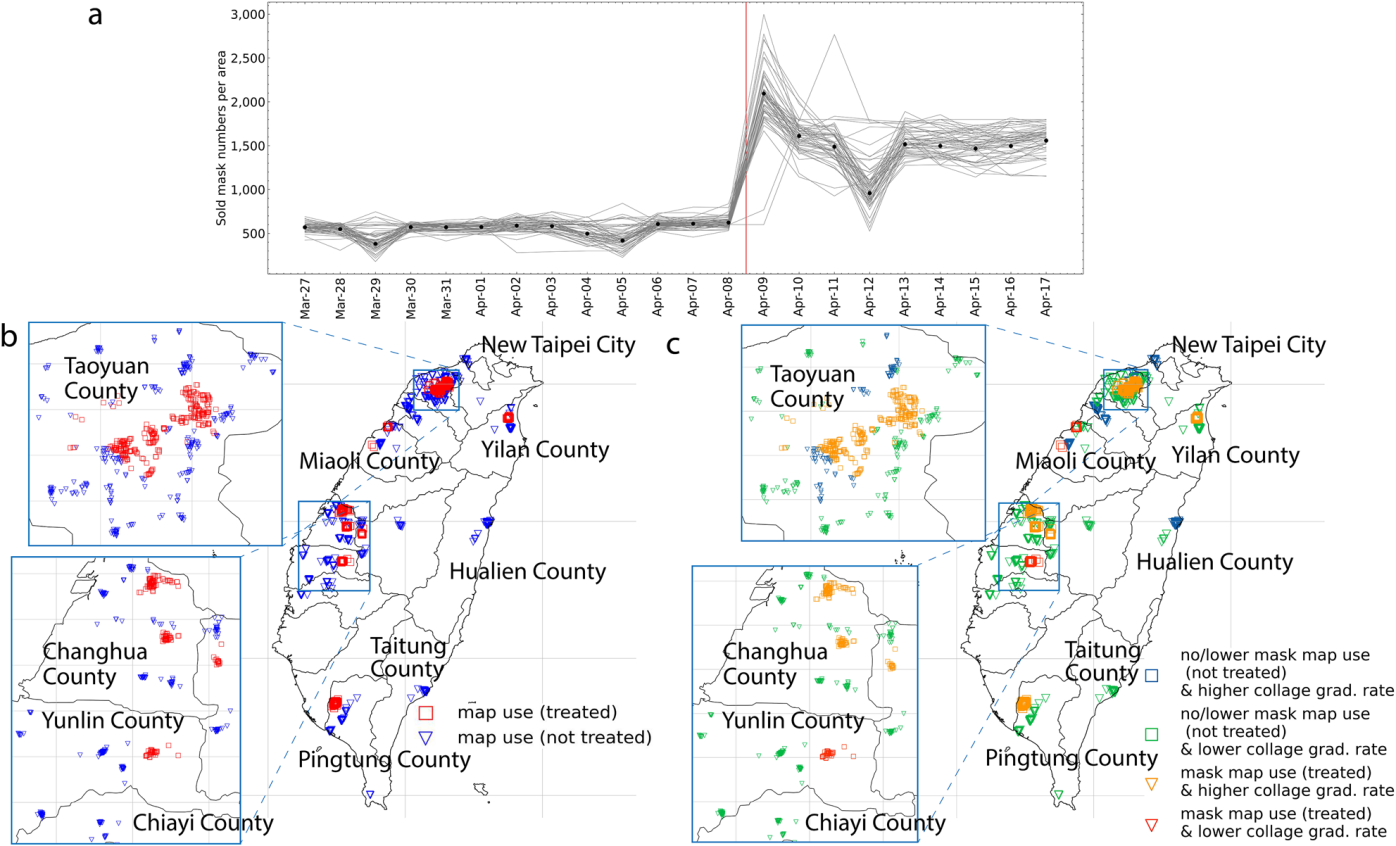
Average sold face mask numbers per area before and after the government loosened the mask purchase policy (Panel (**a**), the red line indicates when the policy was changed). The launch date of the mask map is outside of Panel (a) (the launch was in February 2020). The target mask selling stores’ locations are shown in Panels (**b**) and (**c**). In Panel (**b**), the stores are plotted in red if they locate in the mask map use areas (more than 1 % of mask map use), otherwise blue. In Panel (**c**), stores are further categorized according to the areas’ college graduate rates. Several areas do not show any stores in the panels because there were no corresponding mask map usage data in those areas. Thus, they were excluded from the analysis (see “Methods” section). See Table S2 in Supplementary Materials for area information. The maps of Panels (**b**) and (**c**) were created by the authors with Python 3.10.2. and GADM (https://gadm.org).

This study is designed to measure the impact of the open data use case on citizens’ behaviors. We investigate a Taiwanese open-data initiative in which the national government opened up the store-level face mask stock data to the public, intending to reduce panic buying behaviors during the early stage of the Covid-19 pandemic (see also case description in “Methods” section). Panic buying behaviors have been a worldwide phenomenon and challenge during crises, including the Covid-19 pandemic^49,50^. Various factors, such as excessive and lack of information, anxiety, and fear, lead to panic buying behaviors^49,51–54^. We focus on the case of Taiwan’s countermeasures for mask panic buying behaviors because it is arguably one of the most insightful use cases of open data, due to being highly relevant and urgently needed during the pandemic^55,56^. We examine (RQ1) how the open data initiative impacted citizens’ panic buying behaviors. We hypothesize that the release of the open mask data led to a decrease in panic buying behavior (H1). We set this hypothesis because a number of news articles describe the success of Taiwan’s face mask data release^55–57^, yet no scientific research has been conducted on this point. Further, we analyze (RQ2) how, if at all, the impacts of the open data initiative differ among socioeconomic characteristics. We hypothesize that the reduction in panic buying behavior varied among socioeconomic conditions (H2). We test this hypothesis because digital solutions bring different types of effects on different social and economic groups^58,59^. We assume that open data can be the same case. In particular, areas with a larger portion of higher educated populations may have less tendency to participate in panic buying. On the other hand, we assume that areas having a higher portion of elderly and with new Covid-19 cases may tend to hurry to purchase masks independently of the areas’ mask map use.

We conduct a quasi-experiment approach as a method to retrospectively analyze the quantified impacts of the mask map use on citizens’ behaviors. Namely, we use the difference-in-difference (DiD) approach, widely used to infer the effects of different interventions using observational data^60–64^. A benefit of this approach is that it compares the open data initiative’s effects relative to plausible counterfactuals. The DiD model compares the changes in sold mask numbers (related to the degree of panic buying behavior) between the stores in mask map use areas (treatment group) and those in no/lower mask map use areas (non-treated group) before and after the government loosened the mask purchase policy (increased purchasable mask number per person, enforced on April 9, 2020). Our first analyzed difference is between areas with higher mask map use and areas with no/lower use (Fig. 1b). The second difference is the change in the mask purchase policy. In our analysis, the stores in no/lower mask map use areas (non-treated group) serve as the counterfactual, simulating what would have happened in the treated stores without mask map use. To ascertain that the estimates are not driven by confounding factors^65,66^, we implement several different model specifications with different thresholds for the definition of mask-map use area and two sub-samples (higher college graduate rate areas and lower college graduate areas, see “Methods” section). As Panel a in Fig. 1 shows that the number of sold masks increased drastically when the mask purchase policy was loosened (shown in the red vertical line in the figure). This indicates that people, on average, rushed to buy masks when the number of masks they could purchase increased.

**Table 1:** Baseline model’s estimated coefficients (Eq. 2).

Outcome: sold mask n. per household	All stores		Higher college graduation rate areas		Lower collage graduation rate areas
Treatment threshold	1%	3%	1%	3%	1%
Treated (mask map use)	− 2.079***	− 2.151***	− 0.744*	− 0.861***	− 0.475
	(0.448)	(0.337)	(0.354)	(0.264)	(1.648)
Mobility trend score	− 0.155	− 0.011	− 0.086	− 0.011	11.117***
	(0.412)	(0.419)	(0.070)	(0.078)	(0.115)
Close stores’ mask dispersion	− 0.091	− 0.163**	− 0.032*	− 0.067**	− 0.166*
	(0.057)	(0.066)	(0.024)	(0.030)	(0.086)
Max mask stock level	1.515***	1.515***	0.818***	0.810***	2.185***
	(0.190)	(0.192)	(0.123)	(0.124)	(0.273)﻿
New Covid-19 case	0.256**	0.230***﻿	0.102***﻿	0.078	0.421***
	(0.056)﻿	(0.069)	(0.032)	(0.045)﻿	(0.066)﻿
New Covid-19 case on previous day	0.071**﻿	0.057*	0.033***﻿	0.020	0.122***﻿
	(0.027)	(0.029)﻿	(0.014)	(0.018)﻿	(0.027)
Date fixed effects	Yes	Yes	Yes	Yes	Yes
Adjusted $R^2$	0.752	0.743	0.796	0.793	0.755
Number of obs.	33,160	33,160	17,220	17,220	15,940
Number of areas	55	55	41	41	14

Each column in the table represents separate DiD regression. All stores are included in the analysis in Columns 1–2. The stores only in higher college graduate rate areas are included in Columns 3–4. The stores only in lower college graduate percentage areas are included in Column 5. In this study, “higher college graduation rate areas” is defined as having a college graduation rate higher than the third quartile of all areas’ college graduation rates; otherwise, “lower college graduation areas.” The outcome variable is the daily number of sold mask numbers per household. All models present standard errors clustered at the area level. The treatment threshold designates the percentage of users in the sample who actually used the open-data mask map. Treatment dummy thresholds have been changed to 1% and 3% for robust tests (see the “Methods” section for more information on the thresholds).). $$^{***}$$ $$p<0.01$$, $$^{**}$$ $$p<0.05$$, $$^{*}$$ $$p<0.1$$.

## Results

### Baseline results: overall impacts of the mask map

Table 1 summarizes the baseline results obtained by fitting the DiD model of Eq. (2). Columns 1–2 show that, overall, the mask map usage has a negative effect on the number of sold masks. This indicates that, soon after the government loosened mask purchase restriction, stores in mask map use areas (treated group) experienced less panic buying behaviors than other stores. For a robust check, we also implemented the same equation, Eq. (2), by changing the thresholds of the treatment dummy to 3% (for thresholds definition, see “Methods” section), finding similar results as the baseline model.

Nevertheless, although we include various socioeconomic indicators in the equation, some biases might still exist in the equation. Some socioeconomic characteristics, in particular a higher education level, seem to explain differences in the samples. Education levels tend to be higher in the treatment groups (Table 3). Thus, we further estimate the baseline model with two subgroups separately: stores in higher college graduation rate areas and stores in lower college graduation rate areas. In this study, “higher college graduation rate areas” include areas whose college graduation rates are higher than the third quartile of all areas’ college graduation rates. Columns 3–4 in Table 1 show that the estimations only among higher college graduate rate areas’ stores. Column 5 show that the estimations only among lower college graduate rate areas’ stores. Within higher college graduate rate areas (Columns 3–4), the impacts of the mask map usage still have negative effects, indicating reduced panic buying behaviors in the areas. Within the areas having a smaller portion of citizens who graduated from college (Column 5), the effects are insignificant, indicating no evidence of mask map impacts among lower college graduate rate areas.

**Figure 2:**
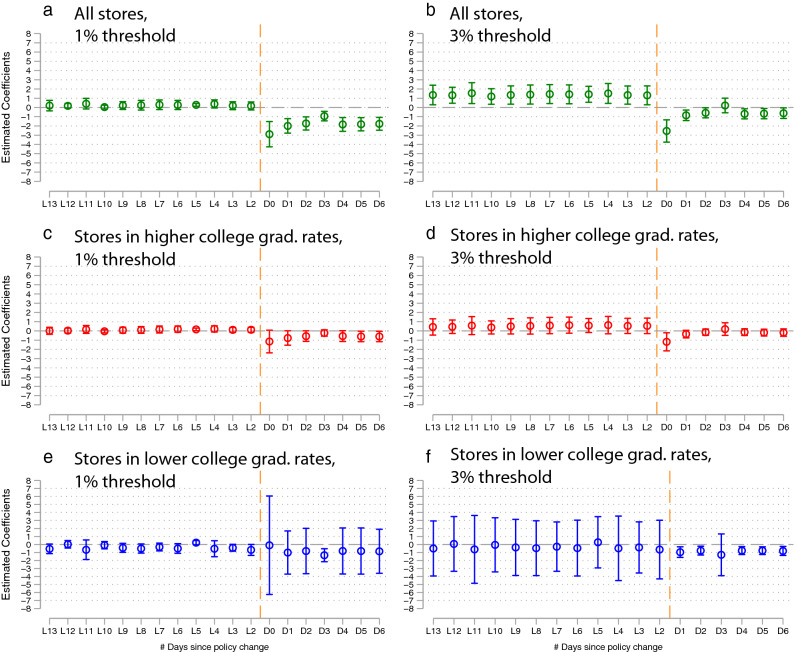
The event study results on the mask map use effects. Each panel represents separate regression using Eq. (3). All panel’s outcome variable is sold mask numbers per household. The estimated coefficients and their 95% confidence intervals are plotted. Vertical orange lines indicate the timing when the government loosened the mask policy. $\hbox {L}k$ and $\hbox {D}k$ in the x-axis represent *k* days before and after the policy change. The dummy variable of the one day before the policy implementation ($k=-1$) is omitted from the regressions. All stores in our samples were analyzed in Panels (**a**) and (**b**). The stores only in the areas with a higher portion of college graduates were analyzed in Panel (**c**) and (**d**). The stores in the areas with the lower portion of college graduates were analyzed in Panel (**e**) and (**f**). The higher college graduation rates areas include the areas whose graduation rates are above the third quartile of all areas’ college graduation rates. Note that for Panel (**f**), D0 (on the first day that the government loosened the mask purchase restriction) has been omitted from the equation for the multicollinearity problem. In the equations, we include various covariates (e.g., maximum mask stock per day per store, new Covid-19 cases per area per day, and college graduate percentage per area). The date fixed effects and standard errors clustered at the area level are included. Full results are presented in Table S4 in Supplementary Material.

We complement these baseline results with the event-study-based analyses. One of the key assumptions of the DiD model is that the treated and the non-treated groups should follow parallel trends in the number of sold masks with the absence of the mask map use. We test this parallel assumption using Eq. (3). We find that the number of sold masks in the treated groups does indeed parallel the number of sold masks in the not-treated group before the policy change, which are reflected around zero estimated coefficients before the policy change (Fig. 2).

Further robust tests were conducted by complementing the baseline model estimations. Namely, we implemented different regressions with non-parametric assumptions. In Table 2, the summary of the Kernel Propensity Score Matching Model estimations is presented. After matching treated and control stores according to their kernel weighted propensity scores based on their baseline characteristics, we still found the impacts of mask map use on reducing sold mask numbers for some estimations (Table 2 Columns 2, 3, and 4). Specifically, the impacts of the mask map use on reducing sold mask numbers among higher college graduate school rates areas were still observed (Rows 3–4 in Table 2). On the other hand, we do not find any impacts of the mask map use on the sold mask number in lower college graduate areas. These results pose further questions on whether the mask map use impacts were only delivered by the digital solution or with other baseline characteristics of stores’ located areas’ socioeconomic conditions.

**Table 2:** Kernel propensity score matching model.

Treatment threshold	All stores		Higher college graduation rate areas		Lower college graduation areas
	1%	3%	1%	3%	1%
Coef. of treatment dummy	− 0.522	− 0.758**	− 1.184***	− 0.720*	1.485
Std.err.	(0.572)	(0.272)	(0.329)	(0.271)	(2.099)
Adjusted $R^{2}$	0.11	0.31	0.44	0.36	0.07
Number of obs.	29,592	15,760	12,202	10,562	12,718

This approach matches treated areas with similar non-treated areas based on observed characteristics, then applies the DiD to the matched areas. Each column in the table represents separate DiD regressions. All stores are included in the analysis in Columns 1–2. The stores only in higher college graduate rate areas are included in Columns 3–4. The stores only in lower college graduate rate areas are included in Column 5. The outcome variable is the daily number of sold mask numbers per household. All models present standard errors clustered at the area level. Treatment dummy thresholds have been changed to 1% and 3% for robust tests (see “Methods” section). $$^{***}$$ $$p<0.01$$, $$^{**}$$ $$p<0.05$$, $$^{*}$$ $$p<0.1$$.

### Socioeconomic differences in the mask map impacts

**Figure 3:**
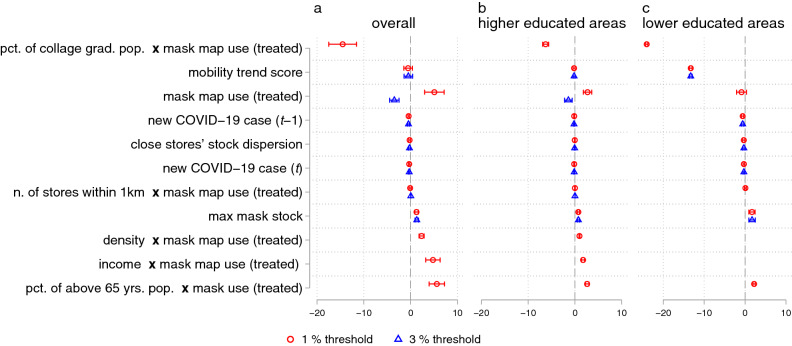
Socioeconomically heterogeneous impacts of mask map use on sold mask numbers. The impact of the mask map on suppressing sold mask amounts is greater when stores are located in the higher college graduate rate areas. This trend was also observed when estimating only with stores in higher graduate rate areas and only with stores in lower graduate rate areas separately. Panels (**a**)–(**c**) show separate DiD regressions (Eq. 4). Panel (**a**) is the estimates with all sample data, Panel (**b**) is the estimates with stores only in higher ratios of college graduates, and Panel (**c**) is the estimates with stores only in lower ratios of college graduates. Dots and bars represent the point estimates and their 95% confidence intervals of the estimations. The variables except dummy variables are standardized to have a mean of 0 and a standard deviation of 1. We use the date fixed effects, and standard errors are clustered at the area level. Full results are presented in Table S3 in Supplementary Materials.

As shown in the statistic summary (Table 3), mask map use areas (treated) seemed to have higher socioeconomic status. As such, a higher percentage of the residents might have been able to work from home; hence they had no need to rush to obtain masks when the mask purchase restriction was loosened. Or, we may assume that these people may have a tendency to use digital solutions, such as the mask map, for better decision-making. On the other hand, the elderly population and those with infected persons near them may have felt at risk more and needed to buy masks as soon as possible. Thus, we further inspect the heterogeneous impacts of the mask map use on sold mask numbers with Eq. (4). By adding the interaction terms between mask map use and other socioeconomic variables, we explore how areas’ basic socioeconomic characteristics are associated with the effects of mask map use. These characteristics include college graduate rates, number of mask selling stores within 1 kilometer of a given store, above 65 years-old population rates, average income, density, and new Covid-19 case numbers. As a result, we observed several strong heterogeneous associations with the impacts of the mask map use on the sold mask numbers (Fig. 3). As indicated by the negative coefficients of the interaction terms between mask map use and higher college graduation rate areas area variables in Fig. 3, the impact of the mask map on suppressing sold mask numbers is greater when stores are located in the higher college graduate rate areas. This trend was also observed when we estimate the same equation with two sub-groups separately: only areas with higher rates of college graduates and only areas with lower rates of college graduates. In contrast, the analysis indicates that the areas with a larger portion of the elderly and the areas with higher average income are more likely to participate in panic buying behavior as the estimated coefficients of interaction terms between the elderly population and mask map use are positive. We did not find any significant impacts of new Covid-19 cases in areas on a given day or the day before on sold mask numbers on the numbers of sold masks.

**Table 3:** Basic statistics of socio-economic variables, describing area characteristics of treated and not-treated stores.

	1% threshold		3% threshold	
	Treated	Not-treated	Treated	Not-treated
Percentage of college graduates	36.26	31.58	36.99	32.96
	﻿(1.81)	(4.92)	(0.00)﻿	(4.71)﻿
Density	2221.44﻿	834.97	2119.26	1275.62
	(1682.50)	(639.44)﻿	(0.00)﻿	(1366.50)﻿
Income	1,023,001	1,014,020	1,182,717	973,590
	(154,196)﻿	(175,436)﻿	(0.00)﻿	(157,568)﻿
Number of stores within 1 km	21.73	15.66	25.68	17.16
	(11.66)﻿	(10.00)﻿	(14.59)﻿	(10.33)﻿
Above 65 year-old population percentage	14.59	15.08	13.01	15.08﻿
	(1.90)﻿	(2.99)﻿	(0.00)﻿	(2.71)

Averages of each variable of each group are shown in the table. The numbers in the parentheses are standard errors. See Figs. S2 and S3 in Supplementary Material for the variables’ distributions. For basic statistics of other variables, see Table S1 in Supplementary Material.

## Discussion

We investigated the impacts of the Taiwan government’s open data initiative providing data on face mask stocks. The initiative intended to help citizens to reduce uncertainty about mask availability while suppressing panic buying behaviors during the early stages of the Covid-19 pandemic. Overall, our analyses supported our first hypothesis, H1, that the data openly provided via the mask map reduced the sold mask numbers when the mask purchase policy restrictions were lifted. Although our data is not a direct observation of panic buying behaviors, the results indicate that the mask map impacted people’s purchasing behavior regarding whether to rush to a store to buy face masks. We can also confirm our second hypothesis, H2, assuming that impacts of digital solutions were limited and varied across socioeconomic conditions of areas: The results suggest that more educated people have changed their behavior after the provision of the open data. We think this might be derived from the fact that more affluent people could work from home in difference from people with a lower socioeconomic status^67,68^. Working from home could result in less urgent demand for face masks. Also, we assume that people with higher socioeconomic status have higher digital literacy being able to use the mask map immediately without any help. This leads us to conclude that people with high digital literacy are more used to making decisions based on data even before the pandemic. Such skills have already been described in the literature as “quantitative map literacy”^69^. Further, government-driven digital solutions tend to be more accepted by people who are concerned about risks and who trust the government^70^. However, in this study, we cannot test the impact of individuals' risks and trust in government, as we cannot access these data. More research is needed to prove the impact of such individual conditions on citizens’ behavior.

The current study contributes to ongoing research on the use of open data for improving pressing matters of common concern in cities and communities. First, the study provides an approach to empirically analyze the impacts of digital solutions in the smart city context. In the last decades, cities have invested in various digital solutions to address issues, yet the assessments of their impacts have not been fully conducted. Our quantitative impact assessments based on a quasi-experiment approach would be applicable for other cases if the data are available. Yet, we emphasize that various perspectives of understanding the impacts based on various types of assessments, including both qualitative and quantitative, are critical due to existing complex impacts. Second, the study’s findings shed light on the importance of understanding the heterogeneous impacts of digital solutions. Smart city constructions, particularly considering during and after the pandemic, has been posed much more challenges and opportunities to develop socio-technological solutions to (re)design better response and recovery in the time of crisis^71,72^. In previous work, lay citizens have been described as a sort of homogeneous group that lacks expertise and knowledge^35,36^. Our study contributes to a more heterogeneous picture of lay citizens showing differences, for example, in their educational level, age, and location of residence. In addition, our concrete case shows a rare scientifically-sound impact of open data on non-expert citizens behavior, which allows more detailed recommendations to open data researchers, and portal developers to improve the use of open data by non-expert citizens. Concretely, we argue that urgency might be an important factor for the public value and usefulness of an open data application, such as data maps. In our case, the pandemic situation and the urgency of becoming prepared for this situation, for instance by buying face masks as a protection were already existing circumstances. The urgency became manifested in terms of citizens’ panic-buying behavior and created the need for information on face masks stocks in local shops. To address this urgent need of information, stock data has been provided openly and a data map has been developed as an information tool. We think that the fact that the data map could be used to directly provide the urgently needed information was the main factor for the value of the face mask data map. One important area of open data research is to better understand citizens’ needs for information^44^. Drawing from the findings of our case study, we would like to encourage designers and developers to, as a first step, seek for potential issues in communities. Only as a second step, developers may seek for open datasets potentially helpful to address an observed need. Identifying more observable primary needs of citizens might be key to better understand less directly observable secondary needs (cf.Wilson, 1981^73^). The case study shows that such community issues might be identified from media coverage and be regarded as primary needs of citizens in more general that potentially can be addressed with information provision (see also “issue of public interest” for civic tech initiatives described by Hamm et al.^33^). If a suitable dataset to address an identified need cannot be found, it is equally important to make lacking datasets explicit and communicate about this observation with the responsible authority. Open data strategies and policies need to be continuously checked whether they still comply with current needs and social change.

This study holds several limitations. First, because we used a quasi-experiment approach, neither field experiments nor a direct observation of mask purchase behavior was undertaken. Due to the lack of access to field data and the difficulty replicating an early pandemic situation, we chose this approach to develop new theories on open data with the help of selected data sets and careful analysis. The results presented in this study might be, beneficial for future field studies, direct observations of behavioral changes, and developments of a prediction model. Secondly, our data did not cover all mask-selling stores because some areas lack the corresponding mask map usage data. Further, our data for the analyses were limited in terms of the time periods: this study could not cover the periods when there was the highest demand for masks and when the civic tech first launched the mask map due to the lack of data availability. Thirdly, this study could not directly observe citizens’ purchase behaviors, stores’ mask selling behaviors, and mask map usage at the individual level. Similarly, as our analyses suggested that the mask map impacts may vary across socioeconomic conditions, further investigation on citizens’ acceptance of digital solutions and factors contributing to their use and the effects of such solutions should be conducted by various approaches. Further investigation on how the impacts of open data and digital solutions, in general, differ among individual levels should be conducted in future research. In particular, it is needed to address the digital divide and the heterogeneous impacts of government and civic-tech solutions on society.

In conclusion, this study examines open data’s impact during the pandemic’s early stage. Our quasi-experiment study shows reduced panic buying behavior as a possible consequence of open publication on store mask stock levels. In addition, this study sheds light on the heterogeneity of the open data’s impacts on citizens. Our study provides a new venue for research on open data by focusing on the actual usage of open data rather than the availability, quantities, or qualities of open data.

## Methods

### Case description

As a precondition, due to the higher demand for yet lower availability of face masks in 2020 in Taiwan, the government fixed the price of face masks at five Taiwan dollars each. Also, people had to show IC-embedded National Health Insurance cards to buy masks, and the purchasable number of masks was restricted. In February 2020, in the early stage of Covid-19, people were flocking to pharmacies to stockpile as many face masks as they could, prompting a spate of panic buying in Taiwan^56^. As a reaction to these events, the freelancer Howard Wu built the first crowdsourcing platform to let people report and query the inventory status for nearby stores on February 4th. He was motivated to build such an open data platform when he saw friends and family messaging up-to-the-minute reports on local stores which would still have masks in stock and which ones would be completely sold out on masks^55^. However, this ad-hoc crowdfunded report system had limitations due to the lack of information in some remote areas and the lack of verification of information posted by the public. This situation led the government to build a system for mask selling stores to report their mask stock level and open each location’s stock levels to the public in early February 2020^74^. Civic-tech initiatives used this open data to make a mask map so that people could check the current inventory of masks so as not to make a trip in vain^74^. With this, citizens only need to open web browsers, and the map would display which pharmacies have how many mask stocks near them. While several mask maps were developed by civic tech initiatives, in this study, we collaborate and utilize the first and the primary one developed by Howard Wu and Audrey Tang (https://kiang.github.io/pharmacies/, accessed 27th July, 2021). This mask map was accessed by about 390,000 people only on the release date and has been accessed by over 3 million unique users. All data do not contain any sensitive personal information and have only been used for academic research under their provision.

### Data

To explore the impacts of Taiwan’s open data policy during the pandemic, we are interested in whether the mask map reduced panic-buying behaviors. Specifically, to capture the degrees of panic buying behaviors, we set daily sold mask numbers at the store level per household as our outcome variable. For a treatment variable, we use the mask map use rate per household in an area. Also, other socioeconomic indicators are used as covariates. In the following subsections, we describe the data used in our analysis.

#### Outcome: sold mask number per store per day

Our empirical analysis uses the store-level daily sold mask numbers per household at 1658 stores in 55 municipal areas in Taiwan. The sold mask number per day was estimated by calculating the gap between the maximum and minimum mask stock levels of a given store on a given day. The original data were published by the Taiwan government as open data. The area-level average sold mask number per day is shown in Panel **a** in Fig. 1. The red vertical line in Panel **a** indicates when the government loosened the mask purchase restrictions. As the figure shows, the sold mask amounts drastically increased after easing the regulation on mask purchases. This trend indicates that people rush to buy masks as soon as the number of purchasable masks increased, as described in several articles^57,58^. Our hypothesis is that this trend of mask purchase surge might have been curbed in the areas where more people used the mask map (see “Introduction” section). To assess this point, this study uses the data before and after the mask purchase limitation was loosened on April 9, 2020 (the purchasable masks rose to 9 masks per adult per two weeks and ten masks for children every two weeks). Our data consists of 21 days (7 days after the policy change and 15 days before the policy change, from March 27 to April 15).

#### Treatment: stores in mask map use areas and others

To test our hypotheses, we divide the areas into two groups: areas with higher mask map usage rates and areas with lower/no mask map usage rates. In doing so, we use the mask map’s daily access numbers based on the records on Google Analytics. Google Analytics is a web analytics service offered by Google that tracks and reports website traffic. This Google Analytics data were provided for this study by the civic tech initiative that developed the mask map. Among Google Analytics data, we use the daily unique access user number per area. In detail, we estimated area-level mask map use rates by calculating mask map unique user numbers divided by household numbers. Then, we categorized the stores into two groups: if a store is in areas where less than 1% of households used the mask map after the policy change, we label the store as in the lower/no mask map use area. If a store is in an area where more than 1% of households used the mask map after the policy change, we label the store as in a higher mask map use area. We link all store locations with Google Analytics area information and exclude such stores that do not match Google Analytics data. Note that users’ browser settings might prevent sharing location information. In such a case, Google Analytics does not count those users in the above user per area metrics. Thus, one percent here does not mean the actual percentage of access but the actual percentage should be larger than 1%. Also, we exclude the bot-like users from the counts. After matching, we have 1658 stores in the analysis sample. 1658 stores are located in 55 areas. Areas’ average number of unique stores is 633.05, and the standard deviation is 609.75 (see Table S2 for each area’s store numbers). In Panel b in Fig. 1, the higher mask map use areas’ stores (treated), and the no/lower mask map use areas’ stores (not-treated) are plotted. For the robust test, we changed the treatment thresholds of two groups to one percent and three percent for each model estimation.

#### Covariates: socioeconomic metrics

It is possible that the two groups, higher and no/lower mask map use areas, have different socioeconomic characteristics, possibly causing bias in the analysis. To address the bias between the treated and non-treated groups, we use the store and area-level socioeconomic factors for the analysis. Using various socioeconomic covariates also contributes to answering if the impacts of the mask map vary among the socioeconomic status of the areas. The covariates included in the analysis are described in Table S1 in Supplementary Material. The area-specific covariates include the percentage of the college graduate population, the percentage of the over 65-year-old population, daily new Covid-19 cases on a given day, daily new Covid-19 cases on one day before a given day, the average density of a given area, and the average income of a given area, mobility trend scores of an area on a given day. For mobility trend scores, we use the Covid-19 mobility trends published by Apple (https://covid19.apple.com/mobility, accessed July 27th, 2021). We also include store-specific covariates: the maximum mask stock level (number of available masks) of a given store on a given day, the number of stores within 1 km of a given store, and the five most closed stores’ average mask stock level (number of available masks) of a given store on a given day. These socio-economic variables were included because digital solutions may have different benefits based on socio-economic characteristics, such as age and educational level^59^. In addition, although these issues on the digital divide are not new, the Covid-19 pandemic highlighted that some populations lack access to digital technology^58,75^. Note that in our analyses, in some equations, some of these covariates were excluded if a covariate has higher correlations with other variables in the same equation (see Table S1).

### Identification strategies

#### Base model

Our identification strategy relies on a difference-in-differences (DiD) approach, a quasi-experiment approach widely used to infer the effects of different interventions using observational data^60–64^. A key advantage of this approach is that it compares the mask map effects relative to plausible counterfactuals.

We focus on before and after loosening the policy on mask purchase regulation. Specifically, the DiD model compares the changes in sold mask numbers between the stores in mask map use areas (treatment group) and the stores in no/lower mask map use areas (not-treated group) before and after the policies were enforced. In other words, the stores in no/lower mask map use (not-treated group) serve as the counterfactual, mimicking what would have happened in the treated stores in the absence of the mask map use.

We present several complementary estimation strategies below, though each is a variant of DiD research design framework. For each store *i* in area *j*, we observe store *i*’s daily sold mask numbers per household in area *j*, $Y_{i}$, and treatment status $D_{it} = 1$ if store *i* in area *j* is treated before time *t* and $D_{it} = 0$ otherwise. The main parameter of interest in DiD applications is the average treatment effect on the treated, $\tau$, which is given by:

1 $$\begin{aligned} \tau \equiv \mathbb {E}[Y_i^{1}(1)-Y_i^{0}(1)|D_i=1] = \{\mathbb {E}[Y_i(1)|D_i=1]-\mathbb {E}[Y_i(0)|D_i=1]\} - \{\mathbb {E}[Y_i(1)|D_i=0]-\mathbb {E}[Y+i(0)|D_i=0]\} \end{aligned}$$

where $Y_i^{D}(t)$ is the potential outcome given treatment $D_i$ at time *t*. Here $t=1$ represents the post-treatment period, $D=1$ represents treatment and $D=0$ represent no treatment. Our base model to estimate such is as the below:

2 $$\begin{aligned} SM_{ijt} = \alpha + \tau (Use_{jt} \times Post_t) + \sum _{h \in H}^{}\beta _{h}avar_h + \sum _{k \in K}^{}\beta _{k}svar_k + \lambda _{t} + \varepsilon _{ijt} \end{aligned}$$

where $SM_{ijt}$ denotes the amount of sold masks at store *i* in area *j* on day *t*. $Use_{jt}$ is the dummy variable indicating whether a shop’s area *j* has a higher mask map usage or not (1 if area *j* is in a higher mask use area; otherwise, 0). Thus coefficient $\tau$, this analysis’s interest, measures the average effect of the mask map usage on the sold mask amounts. $Post_{t}$ is the treatment date dummy (if the day *d* is after the loosening of the mask purchase policy). *avar* is a vector of area-specific socioeconomic variables that are deemed to influence the mask purchase behaviors, including college graduate percentage in an area *j*, above 65 years old population percentage in an area *j*, new Covid-19 case from an area *j* on day *t*, and those of the previous day of day *t*, and mobility trend scores of an area *j* on day *t*. For mobility trend scores, we use the Covid-19 mobility trends published by Apple (https://covid19.apple.com/mobility, accessed July 27th, 2021). Among three available mobility trend metrics (transit, walking, and driving), we only use walking because all three have higher correlations with one another. *cvar* is a vector of store-specific variables, including the maximum mask stock level of a store *i* on day *t*, the five closest stores’ maximum mask stock level dispersion of store *i* on day *t*, and other mask selling store numbers within 1 km of store *i*. $\lambda$ is date fixed effects, and $\epsilon$ is the error term, which we cluster at the area level.

As described in the previous section, we hypothesize that people tend not to do panic buying if they have information about mask stock levels of their nearby stores. Thus, we expect that the estimated value of $\tau$ would be negative. Negative $\tau$ would indicate that a store within an area where more people use the mask map had a smaller increase in sold mask numbers when the government loosened the mask purchase policy compared to other stores with no/lower mask map use areas.

#### Event study

The underlying assumption for the DiD estimator is that treated and non-treated areas would have parallel trends in the number of sold masks in the absence of mask map use. Even if the DiD estimator's results show that sold mask numbers were less when the mask policy was loosened, the results may not be driven by the mask map use rate but by systematic differences in treated and non-treated areas. This assumption is never tested because we cannot observe the counterfactual: what would happen to the sold mask levels if nobody used the mask map. Nevertheless, we can examine the trends in sold mask levels of both treated and non-treated groups before the mask policy was loosened. In doing so, we conduct an event study with the following equation:

3 $$\begin{aligned} SM_{ijt} = \alpha + \sum _{m=k, m\ne -1}^{M} \tau ^{k}\times Use_{jt,k} + \sum _{h \in H}^{}\beta _{h}avar_h + \sum _{k \in K}^{}\beta _{k}svar_k + \lambda _{t} + \varepsilon _{ijt} \end{aligned}$$

where $Use_{jt,k}$ are a set of dummy variables indicating the treatment status at different periods. Here, we investigate 13 days before and 7 days after the mask purchase policy was loosened (April 9th, 2020). The dummy for $m=-1$ is omitted in Eq. (3) so that the mask map use effects are relative to the period one day before the policy change. The parameter of interest $\tau ^{k}$ estimates the effects of the mask map use *m* days before/after the policy changes, testing whether the treatment (mask map use) affects the number of sold masks before loosening the policy. Intuitively, the coefficient $\tau ^{k}$ measures the difference in the number of sold mask amounts between areas with mask map use and otherwise in period *k* relative to the difference one day before the policy change. If the mask map uses reduced sold mask numbers, $\tau ^{k}$ would be negative when $k \ge 0$. If the pre-treatment trends are parallel, $\tau ^{k}$ would be close to zero when $k \le -1$.

#### Socioeconomic heterogeneity

In addition, the robust check for the potential biases is needed even when parallel trends hold^65,66^. The higher use of mask map area may likely already be on very different trajectories from the area that have lower map usages. In Table 3, two groups of areas seem to have different characteristics: the higher map usage area tends to be younger, more populated, and more educated. We may think parallel trends conditional on observables do not hold in this context. This would be the case if the higher mask map use areas’ population would have decided not to go rush to a store to buy a mask, for example, because they can work remotely and stay home, thus not demanding to buy masks immediately.

To address such potential bias, we use several strategies, including adding interaction terms between socioeconomic covariates and treatment dummy, conducting kernel propensity score matching difference-in-difference, and estimating above all models with several subgroups (higher socioeconomic areas only, lower socioeconomic areas only, changing the thresholds of mask used for the dummy variable $Use_{jt}$). First, we add interaction terms between socioeconomic covariates and the treatment dummy as the following specification:

4 $$\begin{aligned} SM_{ijt} = \alpha + \tau (Use_{jt} \times Post_t) + \sum _{h \in H}^{}\beta _{h}(dvar_h\times Use_{jt} \times Post_{i,k}) + \lambda _{t} + \varepsilon _{ijt} \end{aligned}$$

where $\tau$ measures the average effect of the mask map usage on the sold maks amounts, as same as Eq. (2). The difference from the base model (Eq. 2) is $dvar_h\times Use_{jt} \times Post_{i,k}$, the interaction terms between socioeconomic covariates ($dvar_h$) and the treatment dummy variables ($Use_{j,t} \times Post_{i,k}$). This interaction term is 0 if a shop’s area *j* does not has a higher mask map usage or the policy was not implemented yet on a day *t*. With this interaction term, we can investigate if socioeconomic variables ($dvar_h$) have relations with the treatment ($Use_{j,t} \times Post_{i,k}$). For example, if an area with higher education levels strongly relates to the mask map usage, the interaction term’s coefficient $\beta _h$ would be larger or smaller; otherwise insignificant.

Secondly, we also investigate the impact of the mask map usage with kernel propensity score matching DiD approach. This approach is the combination approach between DiD and kernel score matching, treating areas with similar non-treated areas based on observed characteristics, then applying the DiD to the matched areas. Thus, kernel matching works as a counterfactual using all control observations and assigns a positive weight to all observations within the areas only observations in the neighborhood. With the kernel propensity DiD approach, we first estimate the propensity scores (the likelihood of being treated) based on the following equation with a probit model: $p_{i} = \mathbb {E}(D_i=1|X_i)$ where $X_i$ is the same baseline covariates as the baseline model (Eq. 2) for both treated and non-treated groups. Namely, mobility trend of an area *j*, five nearest stores’ mask dispersion, maximum mask stock level of a store *i* on day *j*, daily Covid-19 new case in an area *j* and those of the previous day, number of other mask selling stores within 1 km of store *i*, percentage of college graduates of area *j*, percentage of above 65-year-old in an area *j*, the average income of an area *j*, the density of an area *j*. Then, treated and control stores are matched according to their kernel-weighted propensity scores. The weights are used in Eq. (1) to obtain a kernel propensity score matching DiD treatment effects: $\tau = \mathbb {E}[Y_i(1) - w_i \times Y_i(0)|D_i=1]$. To increase the internal validity of the estimates, the overlapping regions of the propensity for treated and non-treated groups are restricted.

### Ethical statements

Data were analyzed at an aggregate level, and no particular citizens are individually identifiable on the data. No individual has been contacted. All methods were carried out in accordance with relevant guidelines and regulations.

## Supplementary Information

Supplementary Information.

## Data Availability

Data and code presented in this study are available on reasonable request from the corresponding author but restrictions apply to the availability of mask map related data, which were shared only for the current study.
