# Supplementary material for: Do open data impact citizens’ behavior? Assessing face mask panic buying behaviors during the Covid-19 pandemic
Source: Sci Rep. 2022 Oct 20;12:17607. doi: 10.1038/s41598-022-22471-y (PMC9584957; doi:10.1038/s41598-022-22471-y)
Supplement: Supplementary file 1 — Supplementary Information. [file 41598_2022_22471_MOESM1_ESM.pdf]

Supplementary Materials for **Do open data impact citizens' behavior? Assessing face mask panic buying behaviors during the Covid-19 pandemic**

Yuya Shibuya, Chun-Ming Lai, Andrea Hamm, Soichiro Takagi, Yoshihide Sekimoto

A. Covariates, summary statistics, and valance

Histogram of outcome variable and covariates are shown in Figures S2 and S3. The summary statistics between treated and non-treated groups are presented in Table 3 in the main text. The treated group has higher college graduation rates, higher density, and more mask-selling stores close to one another than the non-treated group. To quickly check the parallel trend assumption, we plot the original outcome data of treated and non-treated groups within each subgroup in Figure S1. For the event-study-based test on parallel trend assumption, see Figure 2 and Table S4.

B. Estimated results of equations 4 and 3

We present the estimated results of Eq. 4 and 3 are presented in Tables S3 and S4. The visual presentations of these estimated values are in Figures 3 and 2 in the main text.

C. Short- and medium-term impacts of the mask map use dummies

The effects of loosening the mask purchase restrictions may have persisted only in short-term periods. When the mask policy was changed on April 9, we observed spikes in the sold mask numbers at national levels only for two days or so (Fig. 1). Hence, we expect that the short-term benefits of the mask map were strong. To investigate such short-term effects of demand and mask map use, we fit the following equation:

$$SM_{ijt} = \alpha + \tau_s(Use_{jt} \times ShortPost_t) + \tau_m(Use_{jt} \times MediumPost_t) + \sum_{h\in H} \beta_h avar_h + \sum_{k\in K} \beta_k svar_K + \lambda_t + +\varepsilon_{ijt} \tag{5}$$

where $ShortPost_{jt}$ is the interaction term between whether an area is treated (mask map use at 1 % or 3 % threshold) and the short term post-policy change period (April 9 and April 10). While $ShortPost_{jt}$ is the interaction term between whether an area is treated (mask map use at 1 % or 3 % threshold) and the medium term post-policy change period (April 11 and April 15). Other varaibles are defined in the same way as in Eq. (2). For the statistical test, standard errors are clustered at the area level.

Figure S4 reports our findings (Eq. 5), comparing short-term and medium-term impacts of the mask map. The reduction in overall sold mask numbers was greater among mask map use areas' stores within two days of the policy changes. However, the estimations only among higher college graduate rate areas found smaller impacts of the mask map use on reducing mask purchasing. In addition, we found almost no impacts only within lower college graduate areas' stores. Although overall trends of higher mask map use areas have experienced less panic buying, these findings also suggest that it might have been driven by other socioeconomic conditions, such as education and economic differences, as discussed in the previous subsection.

D. Alternative identification designs

In the main text, we compare stores in mask map use areas (treated areas) and those in no/lower map use areas (not-treated areas). As discussed in the main text, higher college graduation rates have a relation to lower sold mask numbers (reducing panic-buying behaviors) after loosening mask purchase restrictions. Here, to complementary check this trend, we add the dummy variables of higher college graduate rate areas in the DiD model as shown below:

$$SM_{ijt} = \alpha + \tau_{edu}(HighEdu_j \times Post_t) + \tau(HighEdu_j \times Use_{jt} \times Post_t) + \sum_{h\in H} \beta_h dvar_h + \lambda_t + \varepsilon_{ijt} \tag{6}$$

where $HighEdu_j$ is dummy variables of higher college graduates. We test two types of dummy variables of higher college graduate areas: First, we consider areas whose college graduation rates are above the average (33%) of the college graduate percentage as higher college graduates areas. Secondly, we consider areas whose college graduation rates are above 40 % as higher educated areas. For Use_{jt}, we also test two types of thresholds (1% and 3%) as same as the main analysis (Eq. 2).

In addition, we conducted the event-study approach with the higher educated areas as a treatment to test the parallel trend assumption. By following the event study design of Eq. 3, the equation is as the following:

$$SM_{ijt} = \alpha + \sum_{m=k,m\neq -1}^{M} \tau^k \times HighEdu_{jt,k} + \sum_{h\in H} \beta_h avar_h + \sum_{k\in K} \beta_k svar_K + \lambda_t + \varepsilon_{ijt} \tag{7}$$

	Variable	Description	Mean	SD	Min	Max	eq. (2)	eq. (3)	eq. (4)
Outcome	Outcome	number of sold masks per store per day	2.48	3.95	0.00	124.08	✓	✓	✓
Area-specific covariates	Pct. college grad.	percentage of college graduation population of an area	33.32	4.64	18.14	44.14		✓	×
	Pct. of above 65 yrs. pop.	percentage of above 65% population of an area	14.90	2.65	8.17	28.58		✓	×
	Income	average income of an area	1017362.00	167806.70	764655.00	1294404.00		✓	×
	Density	average density of an area	1350.93	1325.67	5.75	5170.78		✓	×
	Mobility trend index	Apple's mobility trend index of an area	100.44	16.55	77.93	132.54	✓	✓	✓
	New Covid-19 case (t)	new Covid-19 cases on a given day in an area	0.08	0.27	0.00	1.00	✓	✓	✓
	New Covid-19 case ($t-1$)	new Covid-19 cases on one day before a given day in an area	0.08	0.27	0.00	1.00	✓	✓	✓
Store-specific covariates	Closest stores' mask dispersion	a store's five closest stores' standard deviation of the mask stock numbers	431.00	354.67	0.00	6439.44	✓	✓	✓
	N. stores within 1km	a number of mask-selling stores within 1km of a given store	17.92	11.04	0.00	56.00			×
	Maximum mask stock level of a store	max mask stock number of an area on a given day	1035.18	798.99	0.00	17877.00	✓	✓	✓

Table S1. Descriptive statistics of variables included in equations. ✓indicates a variable is used in the corresponding equation. × indicates a variable is used in the corresponding equation as an interaction term with treatment variables. Some variables were excluded in some equations due to high correlations with other variables.

where $HighEdu_{jt,k}$ are a set of dummy variables indicating the treatment status at different periods. We investigate 13 days before and 7 days after the mask purchase policy was loosened (April 9th, 2020). The dummy for $m = -1$ is omitted in Eq. (7) so that the higher college graduation impacts are relative to the period one day before the policy change. The parameter of interest τ^k estimates the effects of the higher college graduation rates m days before/after the policy changes, testing whether the alternative treatment (higher college graduation rate) affects the number of sold masks before loosening the policy. Intuitively, the coefficient τ^k measures the difference in the number of sold mask amounts between areas with higher college graduation rates and otherwise in period k relative to the difference one day before the policy change.

As a result, with the alternative model (Eq. 6), we still found that the interaction terms between mask map use and the higher college graduation rate have significant negative impacts on reducing sold mask numbers (Figure S5 and Table S5). This alternative approach also supports that the mask map usage has an impact on reducing panic buying behaviors. The parallel assumption test results are shown in Figure S6 and Table S6.

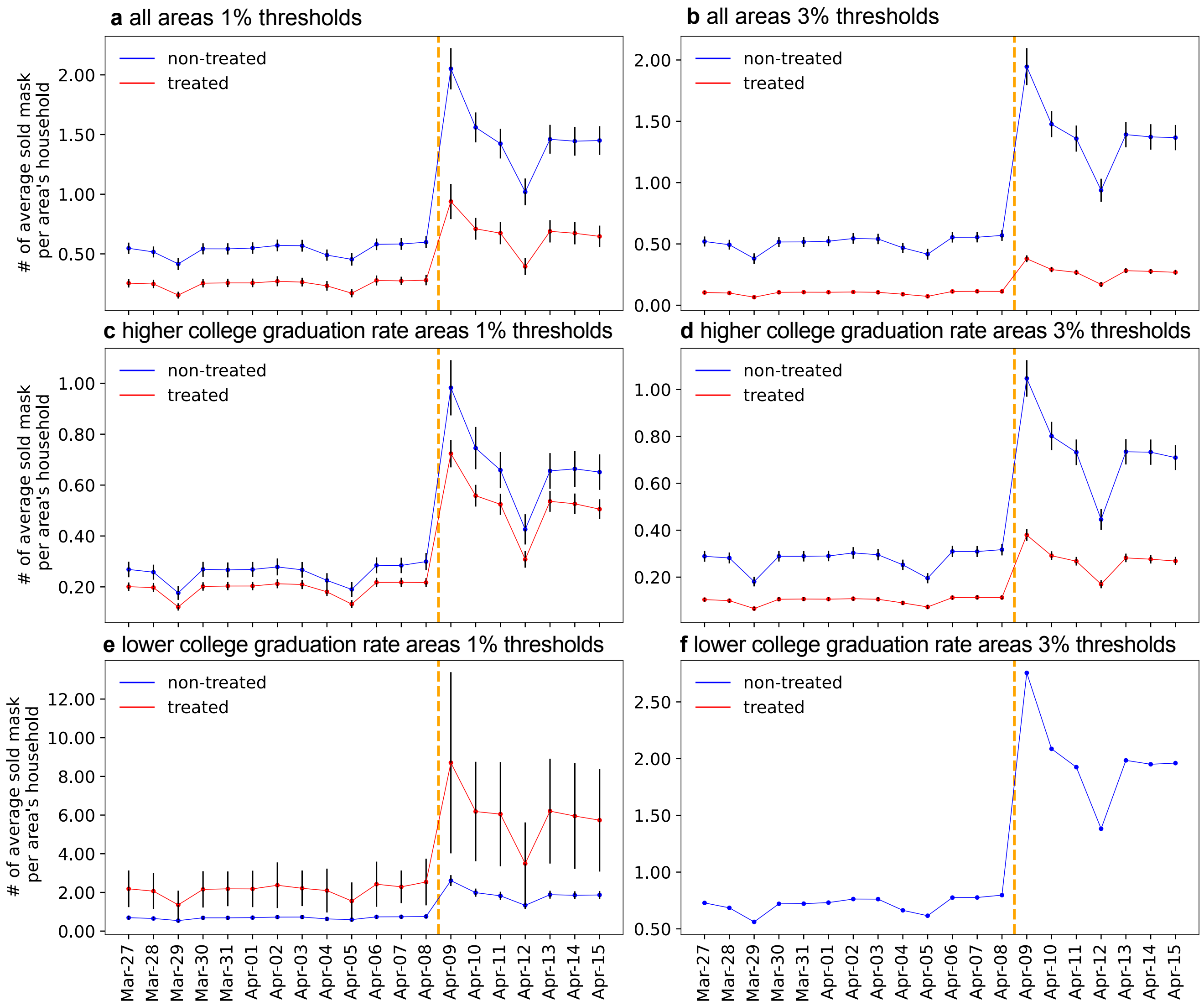

Figure S1. Daily average number of sold masks (Outcome variables). Panels **a-f** represent different subgroups of our samples. Dots and vertical lines are average points and 95 % confidence intervals, respectively. Orange dashed lines show when the government changed the mask purchase restrictions. Panels **a-b** show the outcome of all stores with 1 % (**a**) and 3 % (**b**) thresholds of mask map use definitions, respectively. Panels **c-d** show the outcome of stores only in higher college graduation rate areas. Panels **e-f** show the outcome of stores only in lower college graduation rate aras.

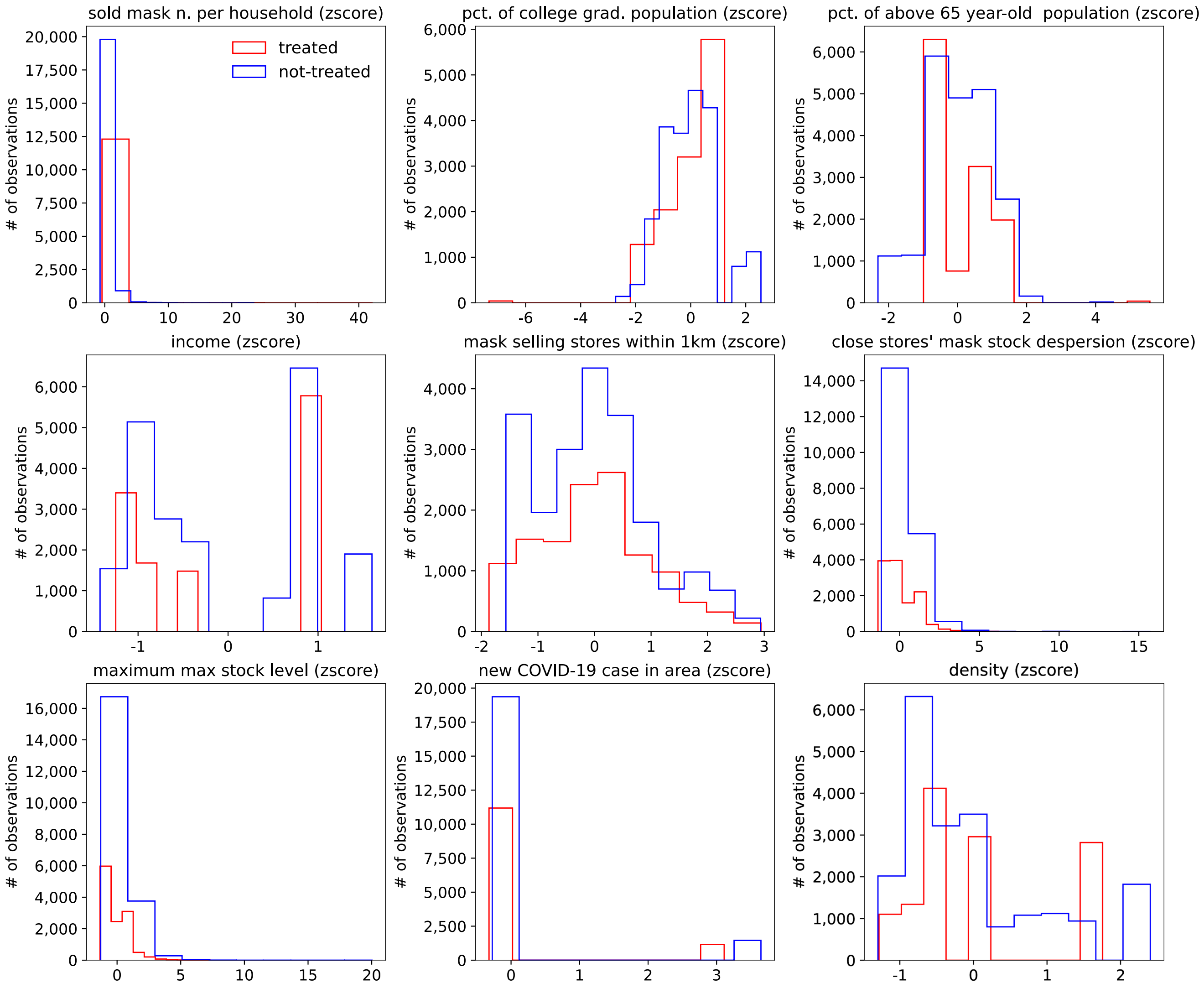

Figure S2. Histogram of outcome and covariates according to treated and non-treated groups in the case of 1 % threshold for the definition of mask map use. Each panel depicts each variable's histograms. Two types of histograms are included in each panel. Histograms in red describe those of treated stores (stores in the mask map use areas), and histograms in blue describe those of not-treated stores (stores outside of the mask map use areas). All histograms are created after converting variables in z-score (mean = 0, standard deviation = 1).

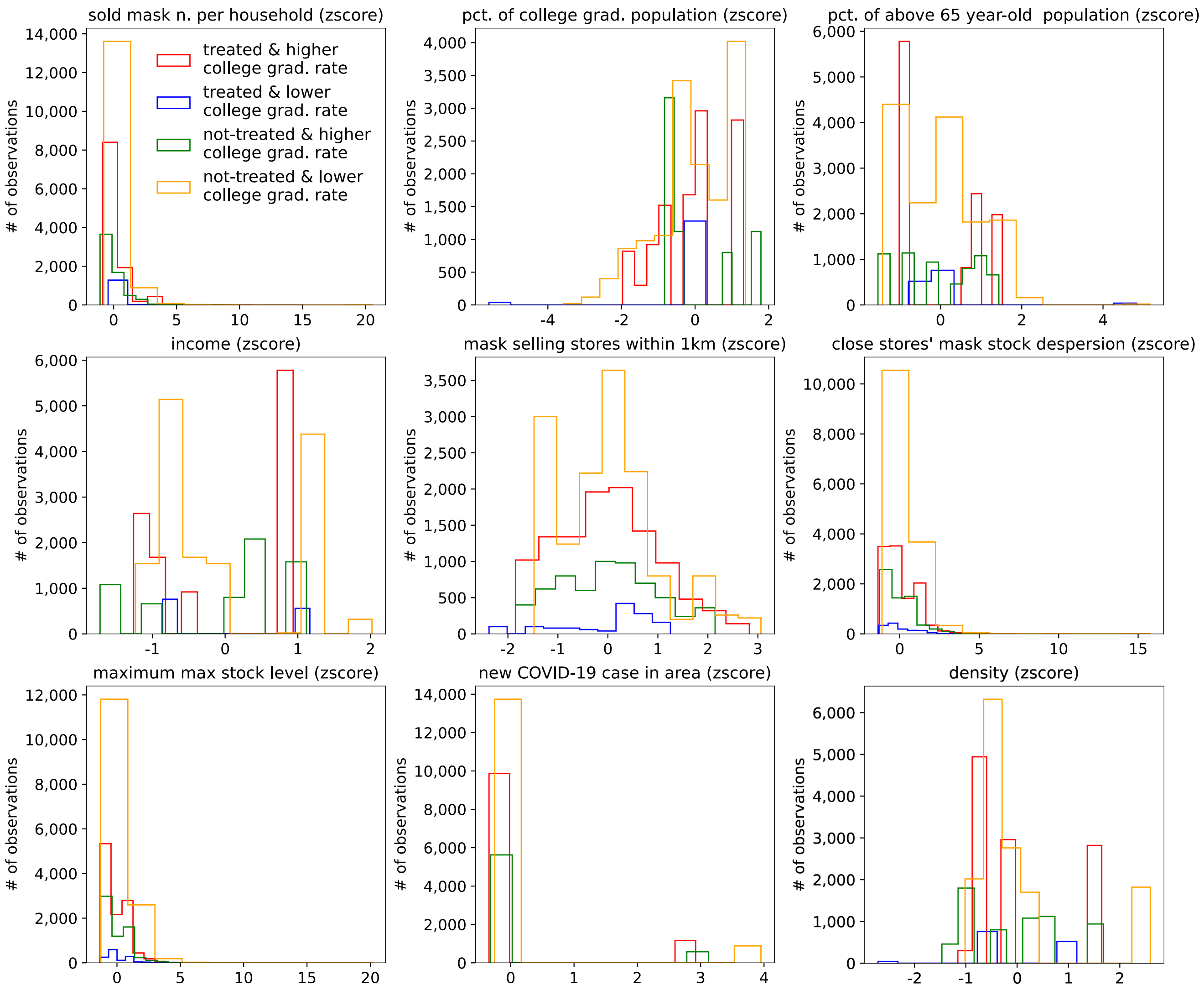

Figure S3. Histogram of outcome and covariates according to treated and non-treated groups (in the case of 1 % threshold for the definition of mask map use) and areas' college graduation rates. Each panel depicts each variable's histograms. Four types of histograms are included in each panel. Red histograms represent those of treated stores (stores in the mask map use areas) in higher college graduate rate areas. Blue histograms those of represent treated stores in lower college graduate rate areas. Green histograms represent those of non-treated stores in higher college graduate rate areas. Orange histograms represent those of non-treated stores in lower college graduate rate areas. All histograms are created after converting variables in z-score (mean = 0, standard deviation = 1).

Area name	Number of mask selling stores	treated (1% threshold)	treated (3%)	higher college grad. rate.
Bade District	1,008			
Beidou Township	336			
Beigang Township	441			
Caotun Township	483			
Changhua City	1,596	✓		✓
Chaozhou Township	546			
Dacun Township	147			
Daxi District	378			
Dayuan District	399			
Donggang Township	378			
Douliu City	798	✓		
Dounan Township	399			
Guanyin District	357			
Guishan District	987			
Hemei Township	462			
Hengchun Township	126			
Hualien City	1,134			✓
Hukou Township	336			
Huwei Township	840			
Jiaoxi Township	147			
Longtan District	588			
Lukang Township	420			
Luodong Township	903			
Luzhu District	1,197			x
Magong City	336			
Mailiao Township	273			
Miaoli City	693			✓
Minxiong Township	546			
Nantou City	315	✓		✓
Neipu Township	252			
Pingtung City	1,764	✓		✓
Pingzhen District	987			✓
Puli Township	609			
Puyan Township	105			
Puzi City	357			
Sanxing Township	42			
Shitan Township	21			
Shoufeng Township	126			
Taitung City	714			
Tamsui District	840			✓
Taoyuan District	2,961	✓		✓
Tianzhong Township	357			
Toufen City	483			
Wulai District	21			
Xihu Township	42	✓		
Xinwu District	105			
Yangmei District	777			
Yilan City	966	✓		✓
Yuanlin City	861	✓		✓
Zaoqiao Township	21			
Zhongli District	3,108	✓	✓	✓
Zhubei City	1176			✓
Zhudong Township	483			✓
Zhunan Township	546	✓		
Zhushan Township	525			

Table S2. Each area's number of stores, treated status, and higher college graduation rate status. In Column 2, ✓indicates the corresponding area is regarded as treated (the mask map use area with 1 % threshold). In Column 3, ✓indicates the corresponding area is regarded as treated (the mask map use area with 3% threshold). In Column 4, ✓indicates the corresponding area is regarded as the higher college graduation rate area (above the third quartile of all areas' college graduation rates). In this study, we excluded areas whose mask map usage data are unavailable. For locations of the stores, see Figure 1.

	overall		higher college graduation rate areas		lower college graduation rate areas	
Treatment threshold	1 %	3 %	1 %	3 %	1 %	3 %
Mask map use (treated)	5.071***	-6.526***	2.700***	-1.974*	-0.877	
	(1.039)	(1.013)	(0.413)	(0.730)	(0.600)	
Mask map use (treated) × pct. college grad.	-14.514***		-6.254***		-24.048***	
	(1.482)		(0.295)		(0.133)	
Mask map use (treated) × n. stores within 1km	-0.095	0.041***	0.004	0.010	0.072	
	(0.110)	(0.010)	(0.032)	(0.006)	(0.099)	
Mask map use (treated) × pct. above 65 yrs. pop.	5.606***		2.609***		2.209***	
	(0.821)		(0.179)		(0.140)	
Mask map use (treated) × income	4.773***	3.853***	1.747***	0.583		20.864
	(0.767)	(0.840)	(0.157)	(0.636)		(17.468)
Mask map use (treated) × density	2.347***	-2.935***	0.976***	-0.893*		-28.419
	(0.271)	(0.491)	(0.046)	(0.316)		(27.680)
Mobility trend index	-0.536	-0.546	-0.185	-0.193	-13.286***	-13.274***
	(0.458)	(0.457)	(0.098)	(0.096)	(0.166)	(0.167)
Closest stores' mask dispersion	-0.215*	-0.196*	-0.031	-0.029	-0.325*	-0.290*
	(0.096)	(0.097)	(0.033)	(0.035)	(0.132)	(0.135)
Maximum mask stock level	1.260***	1.261***	0.737***	0.730***	1.680***	1.727***
	(0.183)	(0.185)	(0.110)	(0.108)	(0.375)	(0.390)
New COVID-19 case (t)	-0.305***	-0.305***	-0.153**	-0.154**	-0.298**	-0.294**
	(0.070)	(0.070)	(0.045)	(0.045)	(0.097)	(0.097)
New COVID-19 case ($t-1$)	-0.422***	-0.423***	-0.155**	-0.157**	-0.594***	-0.575***
	(0.079)	(0.080)	(0.044)	(0.044)	(0.136)	(0.136)
Adjusted R^2	0.381	0.287	0.681	0.639	0.382	0.299
Date fixed effects	Yes	Yes	Yes	Yes	Yes	Yes
Number of observation	33,160	33,160	17,220	17,220	15,940	15,940

Table S3. Estimated coefficients of DiD approach with interaction terms between socioeconomic covariates and the treatment dummy (Eq. 4). Each column is separate DiD model implementation result. Columns 1-2 show those with all stores in our samples. Columns 3-4 show those with stores in the higher college graduation rate. Columns 5-6 show those with stores in the lower college graduation rate. Standard errors are in parentheses under the corresponding coefficients. All models present standard errors clustered at area level. Treatment dummy thresholds have been changed to 1 % and 3 % for robust tests. In addition, we apply each model for subgroups of stores (stores in higher college graduation rate areas and stores in lower college graduation rate areas). Empty cells indicate the corresponding variables were dropped due to high correlations with other variables. For visualization of coefficients, see Figure 3. ***: $p < 0.01$, **: $p < 0.05$, *: $p < 0.1$.

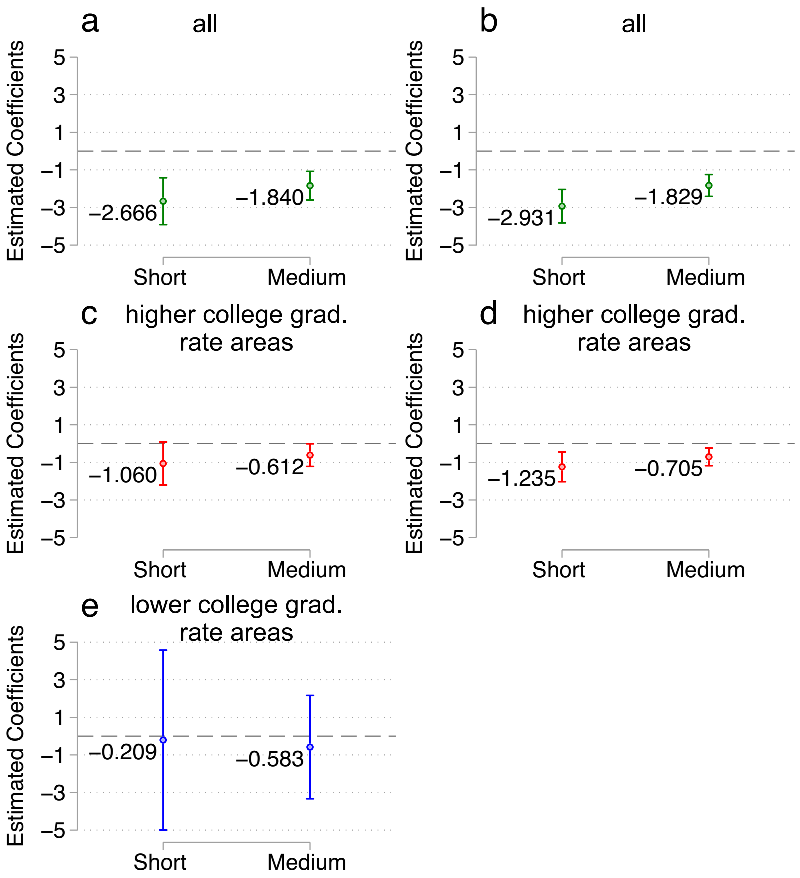

Figure S4. Short- and medium-term impacts of mask map use. The reduction in overall sold mask numbers was greater among mask map use areas' stores within the short term (two days of the policy changes). Panels **a-e** show separate DiD regressions, Eq. (5). Panels **a-b** are the estimated coefficients with all samples. Panels **c-d** are those with only stores in higher portions of college graduates. Panel **e** is those with only stores in lower portions of college graduates. Dots and plots represent the point estimates and their 95 % of confidence intervals for the short-term effects (April 9 and 10) and medium-term effects (April 11 and 15). The number of observations for each regression is 33,160, 33,160, 17,220, 17,220, 15,940 respectively. The date fixed effects are included in each regression. Standard errors are clustered at the area level.

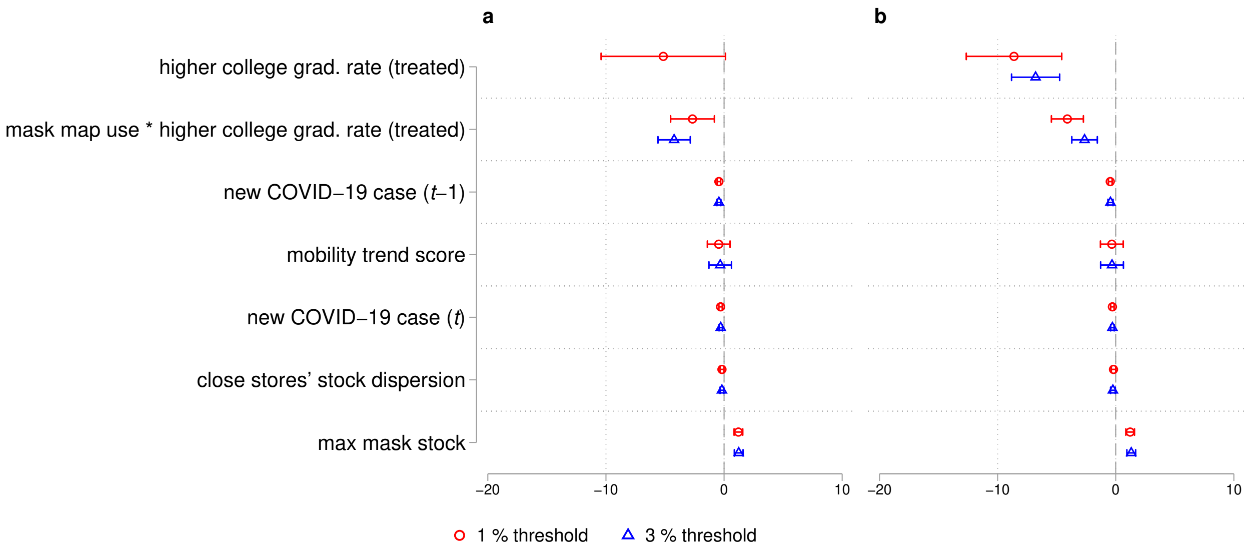

Figure S5. Estimated coefficients of alternative model estimations (Eq. 6). The variables except dummy variables are standardized to have a mean of 0 and a standard deviation of 1. Date-specific effects are included in all the estimations, and standard errors are clustered at the area level. In Panel **a**, the treatment dummy, higher college graduates, is 1 if an area's college graduation rate is above the average (33%). In Panel **b**, the treatment dummy is 1 if an area's college graduation rate is above 40 %. In both panels, red lines are the estimated coefficients when the areas whose mask map use rates are more than 1% are considered as the mask map use areas. The blue lines are the estimated coefficients when the areas whose mask map use rates are more than 3 % are considered as the as mask map use areas. See Table (S5) for the full results.

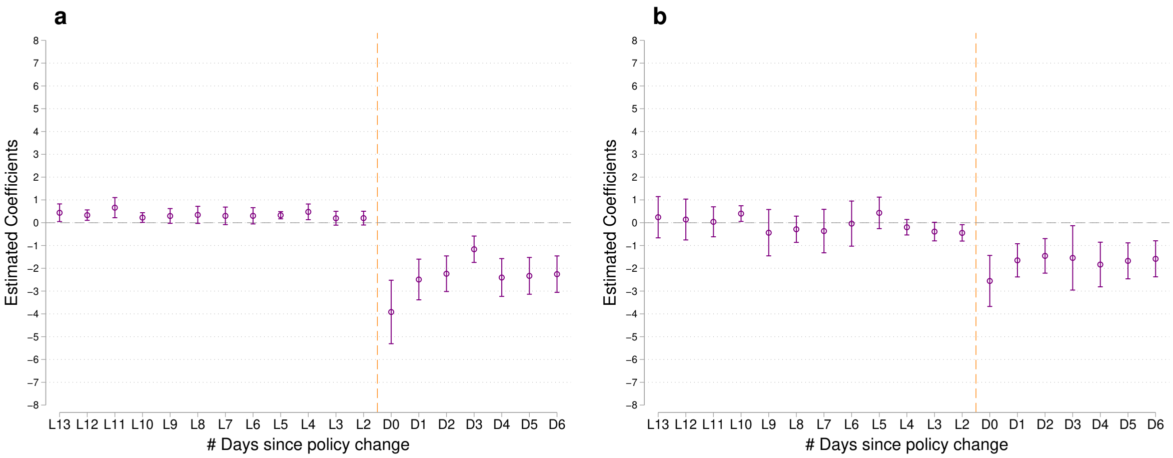

Figure S6. Alternative identification approach's event study model (Eq. 7). Each panel represents separate regression using the event study approach. All panel's outcome variable is sold mask numbers per household. The estimated coefficients and their 95% confidence intervals are plotted. Vertical orange dashed lines indicate the timing when the government loosened the mask policy. Lk and Dk in the x-axis represent k days before and after the policy change. The dummy variable of the one day before the policy implementation ($k = -1$) is omitted from the regressions. Panel **a** shows the estimated coefficients of the Eq. (7) with the treatment threshold as 33%. Panel **b** shows the estimated coefficients of the Eq. 7 with the treatment threshold as 40 %. In the equations, we use various covariates (e.g., maximum mask stock per day per store, new Covid-19 cases per area per day, and college graduate percentage per area). The date fixed effects are included and standard errors are clustered at the area level. See Table S6 for the full results.

	all	higher college grad. rate area	lower college grad. rate area	all	higher college grad. rate area	lower college grad. rate area
threshold		1pct			3pct	
L13	0.204	0.012	-0.543	1.354*	0.433	-0.496
	(0.284)	(0.175)	(0.296)	(0.529)	(0.407)	(1.704)
L12	0.178	0.032	0.027	1.324**	0.453	0.074
	(0.125)	(0.121)	(0.232)	(0.432)	(0.340)	(1.693)
L11	0.409	0.160	-0.659	1.550**	0.577	-0.611
	(0.287)	(0.198)	(0.605)	(0.563)	(0.449)	(2.096)
L10	0.045	-0.039	-0.087	1.188**	0.380	-0.040
	(0.101)	(0.081)	(0.225)	(0.420)	(0.326)	(1.672)
L9	0.209	0.088	-0.413	1.349**	0.498	-0.365
	(0.210)	(0.147)	(0.278)	(0.493)	(0.385)	(1.738)
L8	0.246	0.106	-0.506	1.400**	0.537	-0.458
	(0.262)	(0.169)	(0.282)	(0.516)	(0.408)	(1.695)
L7	0.295	0.158	-0.317	1.448**	0.583	-0.269
	(0.260)	(0.175)	(0.240)	(0.511)	(0.408)	(1.525)
L6	0.267	0.192	-0.499	1.422**	0.624	-0.451
	(0.247)	(0.159)	(0.298)	(0.510)	(0.404)	(1.726)
L5	0.281***	0.167*	0.232	1.426**	0.586	0.279
	(0.075)	(0.064)	(0.130)	(0.431)	(0.352)	(1.584)
L4	0.380	0.221	-0.526	1.519**	0.631	-0.478
	(0.218)	(0.154)	(0.495)	(0.536)	(0.436)	(1.995)
L3	0.190	0.120	-0.413	1.341**	0.542	-0.365
	(0.211)	(0.124)	(0.212)	(0.493)	(0.381)	(1.580)
L2	0.166	0.126	-0.678	1.317*	0.549	-0.631
	(0.215)	(0.125)	(0.340)	(0.509)	(0.390)	(1.813)
D0	-2.893***	-1.147	-0.095	-2.543***	-1.181*	
	(0.682)	(0.568)	(3.046)	(0.600)	(0.453)	
D1	-1.997***	-0.767	-1.006	-0.846**	-0.345	-0.958**
	(0.390)	(0.362)	(1.335)	(0.284)	(0.190)	(0.328)
D2	-1.733***	-0.559	-0.814	-0.583*	-0.138	-0.766**
	(0.356)	(0.265)	(1.400)	(0.271)	(0.161)	(0.272)
D3	-0.935***	-0.226	-1.338**	0.217	0.197	-1.290
	(0.255)	(0.161)	(0.401)	(0.392)	(0.316)	(1.290)
D4	-1.837***	-0.558	-0.811	-0.684*	-0.128	-0.763**
	(0.370)	(0.271)	(1.423)	(0.272)	(0.168)	(0.244)
D5	-1.815***	-0.601*	-0.817	-0.665*	-0.180	-0.770**
	(0.357)	(0.263)	(1.428)	(0.277)	(0.170)	(0.238)
D6	-1.770***	-0.600*	-0.851	-0.620*	-0.179	-0.804**
	(0.349)	(0.261)	(1.361)	(0.285)	(0.185)	(0.276)
Mobility trend index	-0.150	-0.076	11.123***	-0.150	-0.076	11.123***
	(0.419)	(0.063)	(0.119)	(0.420)	(0.069)	(0.120)
Closest stores' mask dispersion	-0.097	-0.036	-0.164	-0.095	-0.033	-0.164
	(0.056)	(0.024)	(0.087)	(0.059)	(0.028)	(0.087)
Maximum mask stock level	1.516***	0.817***	2.186***	1.520***	0.817***	2.186***
	(0.191)	(0.124)	(0.273)	(0.191)	(0.124)	(0.273)
New COVID-19 case (t)	0.266***	0.110**	0.437***	0.278***	0.115**	0.436***
	(0.048)	(0.030)	(0.070)	(0.047)	(0.033)	(0.070)
New COVID-19 case ($t-1$)	0.083***	0.041**	0.128***	0.104**	0.060	0.128***
	(0.014)	(0.011)	(0.028)	(0.039)	(0.030)	(0.030)
Date fixed effects	Yes	Yes	Yes	Yes	Yes	Yes
Adj. R^2	0.754	0.799	0.755	0.752	0.800	0.755
N of .obs	33,160	17,220	15,940	33,160	17,220	15,940

Table S4. Summary of the estimated results of the event study (Eq. 3). Each column represent separate event study results. In Columns 1-3, the results when the thresholds to determine mask map use area is 1 % are shown. In Columns 4-6, the results when the thresholds to determine mask map use area is 3 % are shown. In Columns 1 and 4, all stores in our sample are used. In Columns 2 and 5, the stores in higher college graduation rate areas are used. In Columns 3 and 6, the stores in lower college graduation rate areas are used. Lk is the coefficients of the dummy variables indicating k day before the policy was loosened (April 9, 2020). Dk is the coefficients of the dummy variables indicating k day after the policy change. All models present standard errors clustered at area level. Empty cells indicate the corresponding covariates were dropped due to high correlations with other variables. Standard errors are in parentheses under corresponding estimated coefficients. For the visualization of the results, see Figure 2. ***: $p < 0.01$, **: $p < 0.05$, *: $p < 0.1$.

Alternative treatment threshold (higher college grad. rate areas)	the average (33%)		40 %	
Mask map use area threshold	1%	3%	1%	3%
Mask map use × higher pct. of college grad (treated)	-2.672**	-4.229***	-4.094***	-2.640***
	(0.925)	(0.682)	(0.676)	(0.539)
Higher pct. of college grad (treated)	-5.144		-8.615***	-6.784***
	(2.626)		(2.016)	(1.015)
Mobility trend index	-0.453	-0.327	-0.331	-0.314
	(0.479)	(0.477)	(0.481)	(0.482)
Closest stores' mask dispersion	-0.176*	-0.182*	-0.189*	-0.239*
	(0.084)	(0.08)	(0.094)	(0.1)
Maximum mask stock level	1.215***	1.239***	1.221***	1.313***
	(0.182)	(0.185)	(0.183)	(0.185)
New Covid-19 case (t)	-0.295***	-0.282***	-0.282***	-0.280***
	(0.071)	(0.071)	(0.073)	(0.073)
New Covid-19 case (t-1)	-0.440***	-0.436***	-0.463***	-0.447***
	(0.076)	(0.083)	(0.079)	(0.107)
Adj. R^2	0.341	0.312	0.332	0.243
Date fixed effects	Yes	Yes	Yes	Yes
Number of observations	33,160	33,160	33,160	33,160

Table S5. Estimated coefficients of the alternative model (Eq. 6). Each column show separate DiD estimation result. In Columns 1-2, the results with Eq. (6) when the definition of higher college graduation rate areas is above the average (33 %). In Columns 3-4, the results with Eq. (6) when the definition of higher college graduation rate areas is above 40 %. For visualization of the estimated coefficients, see Figure S5. Standard errors are clustered at the area level. Standard errors are shown in parentheses under the corresponding coefficients. Empty cells indicate the corresponding covariates were dropped due to high correlations with other variables. ***: $p < 0.01$, **: $p < 0.05$, *: $p < 0.1$.

Alternative treatment's threshold (higher college grad. rate)	the average (33 %)	40 %
L13	0.437*	0.241
	(0.193)	(0.451)
L12	0.332**	0.140
	(0.114)	(0.447)
L11	0.660**	0.040
	(0.220)	(0.328)
L10	0.225*	0.399*
	(0.106)	(0.172)
L9	0.296	(0.438)
	(0.162)	(0.507)
L8	0.343	(0.287)
	(0.187)	(0.287)
L7	0.301	(0.367)
	(0.192)	(0.476)
L6	0.303	(0.039)
	(0.178)	(0.494)
L5	0.328***	0.431
	(0.079)	(0.345)
L4	0.476**	(0.198)
	(0.172)	(0.169)
L3	0.196	(0.390)
	(0.152)	(0.204)
L2	0.202	-0.446*
	(0.151)	(0.179)
D0	-3.916***	-2.555***
	(0.695)	(0.558)
D1	-2.492***	-1.651***
	(0.445)	(0.363)
D2	-2.239***	-1.455***
	(0.390)	(0.378)
D3	-1.165***	-1.543*
	(0.289)	(0.704)
D4	-2.404***	-1.833***
	(0.414)	(0.489)
D5	-2.332***	-1.673***
	(0.402)	(0.394)
D6	-2.257***	-1.584***
	(0.399)	(0.394)
Mobility trend index	0.393	0.660
	(0.397)	(0.559)
Closest five stores' mask stock dispersion	-0.116*	-0.160*
	(0.050)	(0.066)
Maximum stock level	1.481***	1.512***
	(0.191)	(0.193)
New Covid-19 case (t)	0.232***	0.321***
	(0.051)	(0.059)
New Covid-19 case ($t-1$)	0.070***	0.092***
	(0.017)	(0.019)
Date fixed effect	Yes	Yes
Adj. R^2	0.768	0.739
N	33,160	33,160

Table S6. Alternative identification approach's event study model (Eq. 7). Lk is the coefficients of the dummy variables indicating k day before the policy was loosened (April 9, 2020). Dk is the coefficients of the dummy variables indicating k day after the policy was loosened. For visualizing the estimated coefficients of the dummy variables, see Figure S6. Standard errors are clustered at the area level. Standard errors are shown in parentheses under the corresponding coefficients. ***: $p < 0.01$, **: $p < 0.05$, *: $p < 0.1$.
